# Supplementary material for: Comparative Study of the Effect of Radiation Delivered by Lutetium-177 or Actinium-225 on Anti-GD2 Chimeric Antigen Receptor T Cell Viability and Functions
Source: Cancers (Basel). 2023 Dec 30;16(1):191. doi: 10.3390/cancers16010191 (PMC10778389; doi:10.3390/cancers16010191)
Supplement: Supplementary file 1 [file cancers-16-00191-s001.zip › cancers-2796383-supplementary.pdf]

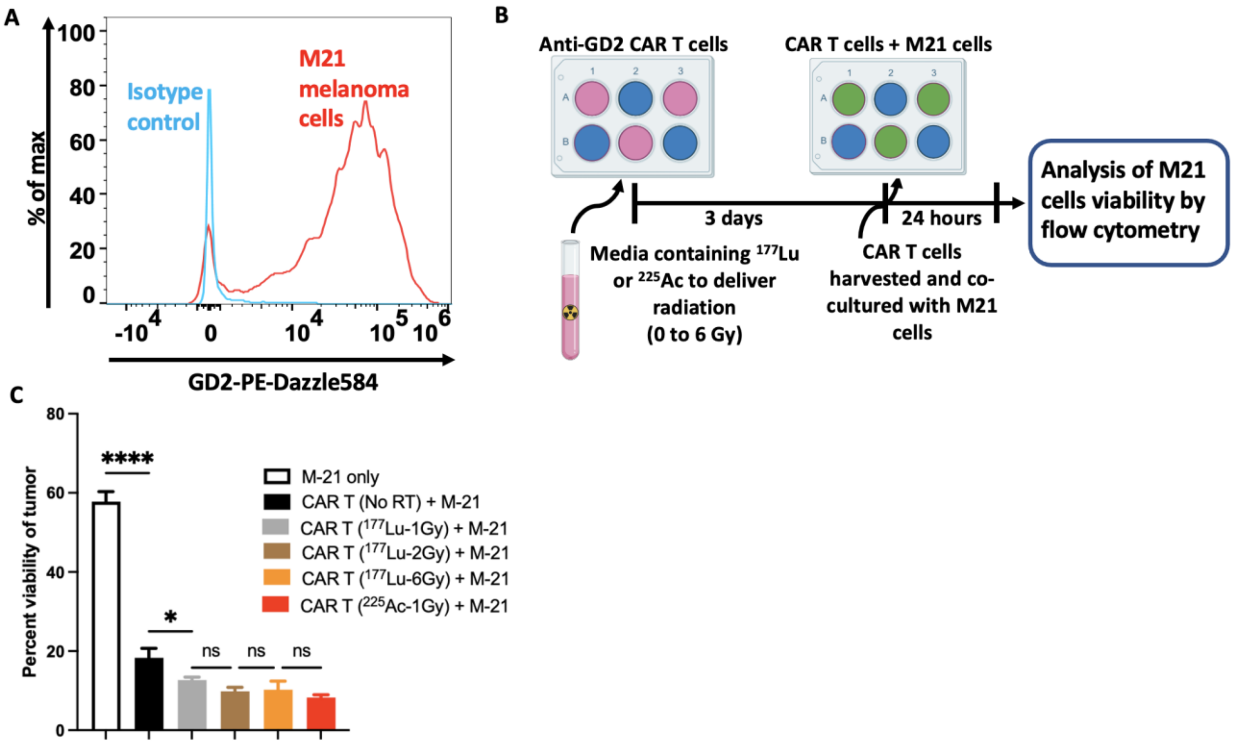


**Supplemental Figure S1: $^{225}$Ac and $^{177}$Lu enhance the cytotoxicity of anti-GD2 CAR T cells against the GD-2 expressing melanoma cell line M21.** (A) The human melanoma cell line M21 expresses GD2. (B) Experimental scheme: After irradiation of CAR T cells by $^{225}$Ac or $^{177}$Lu, the CAR T cells were harvested, washed and trypan blue viability assay was performed. The viable CAR T cells after irradiation were co-cultured with the GD2-expressing human melanoma cell line M21 for 24 hrs at a E:T ratio of 10:1 (Viable anti-GD2 CAR T cells: tumor cells). The viability of the GD2-expressing M21 cells was analyzed by flow cytometry. (C) The exposure of anti-GD2 CAR T cells to radiation delivered by radionuclide enhances their cytotoxicity against M21 cells in a dose-independent manner and irrespective of the type of radionuclide for the combinations tested. One-way ANOVA with Tukey's multiple comparisons correction *= 0.02; ****<0.0001; ns: not significant. Each bar represents the mean (triplicate measurements) and error bar represents the standard deviation.
